# CD16^+^ monocytes are involved in the hyper-inflammatory state of Prader-Willi Syndrome by single-cell transcriptomic analysis

**DOI:** 10.3389/fimmu.2023.1153730

**Published:** 2023-05-11

**Authors:** Yunyun Xu, Xu Hou, Honglin Guo, Zhenyu Yao, Xiude Fan, Chao Xu, Guimei Li, Yanzhou Wang, Yan Sun, Ling Gao, Yongfeng Song, Jiajun Zhao

**Affiliations:** ^1^ Department of Endocrinology, Shandong Provincial Hospital, Shandong University, Jinan, China; ^2^ Department of Endocrinology, Shandong Provincial Hospital Affiliated to Shandong First Medical University, Jinan, China; ^3^ Shandong Clinical Research Center of Diabetes and Metabolic Diseases, Jinan, China; ^4^ Shandong Key Laboratory of Endocrinology and Lipid Metabolism, Jinan, China; ^5^ Shandong Prevention and Control Engineering Laboratory of Endocrine and Metabolic Diseases, Jinan, China; ^6^ Stem Cell Research Center, Shandong Provincial Hospital Affiliated to Shandong First Medical University, Jinan, China; ^7^ Department of Pediatrics, Shandong Provincial Hospital Affiliated to Shandong First Medical University, Jinan, China; ^8^ Department of Pediatric Orthopedics, Shandong Provincial Hospital Affiliated to Shandong First Medical University, Jinan, China; ^9^ Scientific Research Center, Shandong Provincial Hospital Affiliated to Shandong First Medical University, Jinan, China

**Keywords:** Prader-Willi Syndrome, inflammation, CD16+monocytes, single-cell RNA sequencing, mass cytometry

## Abstract

**Background:**

Patients with Prader-Willi syndrome (PWS) have a reduced life expectancy due to inflammation-related disease including cardiovascular disease and diabetes. Abnormal activation of peripheral immune system is postulated as a contributor. However, detailed features of the peripheral immune cells in PWS have not been fully elucidated.

**Methods:**

Serum inflammatory cytokines were measured in healthy controls (n=13) and PWS patients (n=10) using a 65- multiplex cytokine assays. Changes of the peripheral immune cells in PWS was assessed by single-cell RNA sequencing (scRNA-seq) and high-dimensional mass cytometry (CyTOF) using peripheral blood mononuclear cells (PBMCs) from PWS patients (n=6) and healthy controls (n=12).

**Results:**

PWS patients exhibited hyper-inflammatory signatures in PBMCs and monocytes were the most pronounced. Most inflammatory serum cytokines were increased in PWS, including IL-1β, IL-2R, IL-12p70, and TNF-α. The characteristics of monocytes evaluated by scRNA-seq and CyTOF showed that CD16^+^ monocytes were significantly increased in PWS patients. Functional pathway analysis revealed that CD16^+^ monocytes upregulated pathways in PWS were closely associated with TNF/IL-1β- driven inflammation signaling. The CellChat analysis identified CD16^+^ monocytes transmitted chemokine and cytokine signaling to drive inflammatory process in other cell types. Finally, we explored the PWS deletion region 15q11–q13 might be responsible for elevated levels of inflammation in the peripheral immune system.

**Conclusion:**

The study highlights that CD16^+^ monocytes contributor to the hyper-inflammatory state of PWS which provides potential targets for immunotherapy in the future and expands our knowledge of peripheral immune cells in PWS at the single cell level for the first time.

## Introduction

Prader-Willi syndrome (PWS) is a rare, complex, multisystem syndrome with an estimated prevalence of 1 in 10,000–30,000 live births (1), and it was first reported by Prader in 1956. Genetically, PWS is an imprinted disease caused by the lack of active genes located in the paternal chromosome 15q11–q13 region (2). The absence of gene expression in this region mainly occurs through three mechanisms: paternal deletion of the 15q11–q13 region (65–75%), maternal uniparental disomy 15 (20–30%), or imprinting defects (1–3%) (3, 4). The syndrome exhibits a wide clinical presentation spectrum, including hypotonia, developmental delays, cognitive disability, psychiatric phenotypes, sleep disordered breathing and obesity.

PWS patients are at a greater risk for cardiovascular disease and diabetes compared with weight-matched obese controls, which contributes to the most common causes of mortality in PWS (5–9). Furthermore, it was revealed that these comorbidities usually occur at relatively young ages in PWS (10, 11).

These comorbidities were related to chronic inflammation and few studies demonstrated that PWS was associated with increased concentrations of circulating markers of inflammation, such as tumor necrosis factor (TNF) (12), interleukin-6 (IL-6) (13), interleukin-1β (IL-1β) (14), and C-reactive protein (CRP) (13, 15) compared to those with non-syndromic obesity. And these circulating inflammation markers were linked with certain immune cell activation markers (16). It is proposed that the peripheral immune system is activated in PWS, which leads to systemic inflammation manifested by increased cytokine levels and seems to play a critical pathogenic role in the development of these inflammation-related comorbidities. However, detailed characteristics of the peripheral immune cells in PWS have not been fully clarified.

Single-cell RNA sequencing (scRNA-seq) offers an unbiased, comprehensive approach to define cell types and states based on their individual transcriptome and is widely used to reveal immune cell heterogeneity and diversity (17). In this study, we studied the cellular landscape of PWS peripheral immune cells at single-cell resolution *via* scRNA-seq.

## Materials and methods

### Subjects

PWS patients and healthy individuals were included in this study. All study subjects were recruited in Shandong Provincial Hospital affiliated to Shandong First Medical University. Written informed consent was obtained from parent or legal guardian. The diagnosis of PWS had been confirmed by genetic testing and none of the subjects were taking any medications. None of the subjects had a history of cancer, autoimmune disease, diabetes, infections or steroid usage. Serum samples were collected and stored at −80 °C until use.

### Cytokine assay

Serum cytokines were measured for each subject using a 65- multiplex cytokine assays (Cat. No. EPX650-16500-901) on the Luminex 200 system performed at the Laizee Biotech, Shanghai, China. IL-1β levels were measured using high-sensitivity ELISA kits by R&D Systems (Catalog Number HSLB00D) following the instructions exactly.

### PBMCs collection and single-cell RNA-seq

The PBMCs were isolated within 2 hours from fresh EDTA anticoagulated whole blood by density gradient centrifugation using Histopaque-1077 (Sigma, A6929). The single-cell library preparation in our research relied on an available droplet method, the 10x Genomics Chromium Controller. All samples in our study were not pooled. Single-cell RNA-seq libraries were constructed using the Chromium Single Cell 3′ Library & Gel Bead Kit v3·1 (10x Genomics, Pleasanton, CA) according to the manufacturer’s instructions. Cells were divided into gel beads-in-emulsions (GEMs) at nanoliter scales. Then, reverse transcription was used to produce the cDNA. Consequently, each cDNA molecule contained a cell barcode and unique molecular identifier (UMI). We constructed and sequenced libraries at a depth of approximately 100,000 reads per cell by using the Novaseq 6000 platform (Illumina, San Diego, CA).

### Single-cell RNA-seq mapping and pre-processing

The raw sequencing data were converted into fastq format using mkfastq (cellranger 10X genomics, v4.0.0). As soon as the reads were de-multiplexed, they were aligned with the human reference genome (GRCh38; 10x cellranger reference GRCh38 v3.0.0) to obtain the feature-barcode matrices. Then, Cellranger aggr was used to aggregate multiple libraries by default parameters. Seurat R package v4.1.0 was used for subsequent analysis (18). For further analysis, only cells expressing > 800 genes and < 10% mitochondrial genes were included. Doublets and red blood cells were both excluded in downstream analysis. We normalized gene expression for each cell based on the total number of transcripts and log transformation. In order to integrate different datasets, top 2000 highly variable genes for each dataset were recognized *via* using the function FindVariableFeatures with vst method in Seurat (19). Next, samples were integrated with canonical correlation analysis based on the top 20 canonical correlation vectors. The integrated data were scaled and principal component analysis (PCA) was executed. At last, with Seurat’s FindClusters function (0.6 resolution), unsupervised clustering was performed and the cells were visualized by uniform manifold approximation and projection (UMAP). For the rest settings, we used default values for the scRNAseq clustering analysis and the UMAP visualization.

### Differentially expressed genes analysis and functional enrichment analysis

Based on the Wilcoxon-test method implemented in the FindAllMarkers function of Seurat package, we analyzed differential expression genes between two groups. Upregulated DEGs were identified according to the following criteria: (1) a logfold change > 0.25, and (2) p-value < 0.05. To find the function of upregulated genes, we used the function clusterProfiler (version 4.0.5) of the R package. We also used Enrichr, the software for gene set enrichment analysis (GSEA) was used for LINCS L1000 dataset to examine potential biological functions for lists of genes (20).

### Cell-cell communication

Cell-cell interactions analysis was conducted using CellChat (version 1.1.2). On the basis of data from scRNA-seq, the CellChat analysis was performed to infer intercellular and intracellular crosstalk between assigned types of cells. For this analysis, we analyzed each group separately and then used the mergeCellChat function to compare differences between two groups. More details about the package can be found in the previous publication (21).

### Weighted gene co-expression network analysis

The WGCNA package (version 1.70) was utilized to generate modules for co-expression. We applied a soft threshold power of six to calculate the adjacency matrix. Then, the adjacency matrix was transformed into a topological overlap matrix (TOM) to construct a gene tree by hierarchical clustering. We merged modules at a cut height of 0.25 and set the minimum module size to ten. To identify modules correlated with clinical traits, Spearman’s rank correlation coefficients were measured between the different clinical parameters and module eigengenes.

### Gene set score calculation

The AUCell package (1.14.0) was used to calculate gene set scores. For the parameters default settings were used. Inflammation scores were calculated based on the gene set obtained from the Molecular Signatures Database (MSigDB) (GO:0002864). 15q11–q13 gene scores were calculated based on the gene set which contained all genes in 15q11–q13 region. PWS signature score in each cell type was calculated based on the gene set which consist of upregulated genes in PWS of cell types.

### Hierarchical clustering of different gene expression among disease groups at cell type resolution

We calculated the differential gene expression among obese PWS and obese controls relative to normal weight control. We found distinct transcriptional signatures between obese PWS and obese controls in major cell types and calculated the Pearson correlation coefficient using the above transcriptome characteristics. Hierarchical clustering analysis was performed based on the PCC (22).

### Metal-labeled antibodies

All the antibodies were purchased from BioLegend. A series of antibodies used are listed below: antibodies against CD3 (Cat# 300402, RRID: AB_314056), CD4 (Cat# 300502, RRID: AB_314070), CD8 (Cat# 301002, RRID: AB_314120), CD45 (Cat# 304002, RRID: AB_314390), CD14 (Cat# 301802, RRID: AB_314184), CD16 (Cat# 302002, RRID: AB_314202), CD56 (Cat# 318302, RRID : AB_604092), Tumour necrosis factor α (TNF-α) (Cat# 502902, RRID : AB_315254), IL-1β (Cat# 511605, RRID : AB_2861040). According to Fluidigm’s recommendations, antibody conjugations were prepared using the Maxpar Antibody Labeling Kit (Fluidigm, South San Francisco, CA). Metal-labelled antibodies were stored at 4°C at 0.5 mg/mL in PBS-based Antibody Stabilizer (Candor Bioscience).

### CyTOF data acquisition and analysis

PBMCs (∼3x106 cells) were spun (300 g, 5 min) and resuspended in calcium magnesium-free phosphate buffered saline (PBS). Mix well and incubated in 1 mL of 5 μM cisplatin (Fluidigm) at room temperature for 5 min. PBMCs incubated with metal-labeled antibodies followed by Ir-intercalator staining. The PBMCs were washed three times by staining buffer. In the next step, EQ beads were mixed 1:10 with MilliQ water solution to adjust cell concentration to 10^6^ cells/ml. The QE beads are used in order to test if the nebulizer is lined up correctly, by determining the number of events/beads that pass through in a certain amount of time. Before to get normalized data, we calibrated the Helios CyTOF (Fluidigm, South San Francisco,CA). We used Cytobank software to gate the output FCS files to ruled out fragments, dead cells and doublets. Finally, data was clustered and represented in t-SNE maps using the R package Cytofkit (version 1.4.8). Cells were merged by “ceil” in mergeMethod fuction in Cytofkit and the “fixedNum” is 2000. As the mass cytometry data were nonlinear, cytofAsinh was used for data normalization. Data were clustered using the PhenoGraph.

### Statistical analyses

The data were analyzed using SPSS 22.0 or R 4.1.0. The distribution normality was tested using the Shapiro–Wilk test normality test. The Mann - Whitney rank-sum test was used for data with non-normal distribution and the t - test was used for data with normal distribution. Categorical variables were compared by χ2 or Fisher’s exact tests. Multiple linear regression analyses between variables were done using SPSS software (SPSS, Chicago, IL). Regression analyses with the inflammation scores as the outcome predicted by variables BMI, age and PWS scores. Default settings were used for the analysis. We have performed mediation analysis using the bootstrapping method (23) in the ‘mediation’ package in R (version 4.5.0) to test whether the relationship between PWS score and inflammation scores, is mediated by the level of CD16^+^ monocyte. Two thousand bootstraps were run to estimate the confidence intervals. The remaining settings were set default.

## Result

### Serum proinflammatory cytokine levels were elevated in PWS

**Figure 1:**
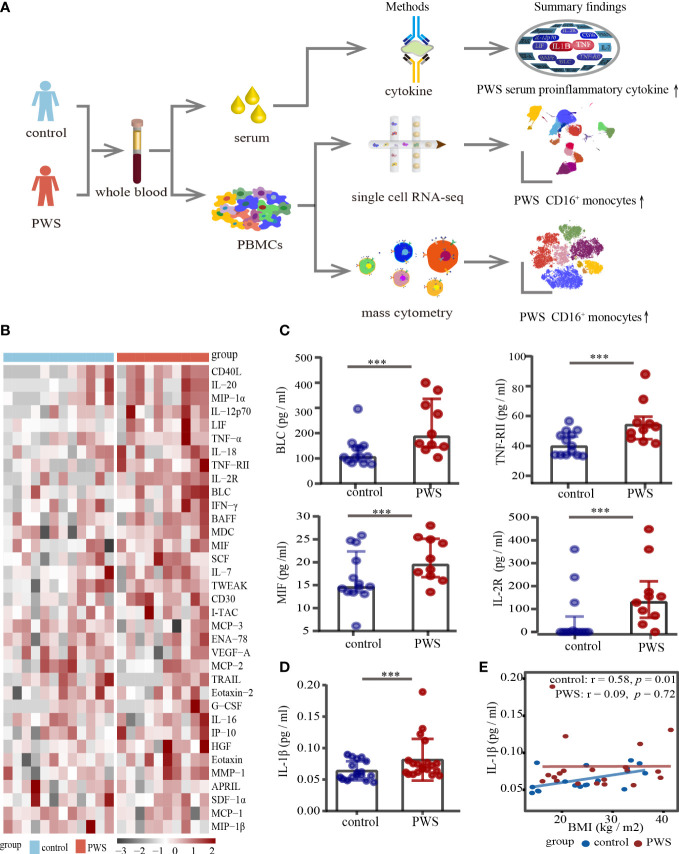
Analyses of serum cytokine levels. **(A)** Study overview. **(B)** Heatmap of all cytokines measured in the PWS (n=10) and control groups (n=13). On the x axis, samples are arranged by the study group and on the y axis, cytokines are displayed according to hierarchical clustering. Cytokines are expressed as log (pg/ml), with black to red colors representing lower to higher expression, respectively. **(C)** Dot plots of cytokines in the PWS group (n=10) compared with the control group (n=13). *p*-value is calculated by the Mann-Whitney U test for comparisons. ***p < 0.05. **(D)** Dot plots of serum IL-1β in the PWS group (n=20) compared with the control group (n=17). *p*-value is calculated by the Mann-Whitney U test for comparisons. ***p < 0.05. **(E)** Spearman correlation between IL-1β serum levels and BMI in the two groups.

In several studies, it has been reported that patients with PWS have elevated levels of serum inflammatory markers (14, 24), however, the data are limited and conflicting. We applied a multi-omics approach in the study (**Figure 1A**) and to determine typical proinflammatory cytokines levels in PWS patients, cytokines of 10 PWS patients and 13 controls were measured using a 65- multiplex cytokine assays. There was no significant difference in gender and body mass index (BMI) between the two groups (**Supplementary Table 1**). Of the 65 indicators, 35 were detectable with 60% of values

in the detectable concentration range for the kit in one of the groups. Interestingly, 19 of the 35 indicators (54%) in the PWS group were 1.2-fold higher relative to the control group (**Figure 1B**; **Supplementary Table 2**). Most of the increased indicators in PWS were proinflammatory cytokines and chemokines, including TNF-RII, IL-12p70, MIF, MIP-1α and TNF-α (**Figure 1B**). Due to the limited number of the study participants, only 5 indicators were statistically higher in PWS (**Figure 1C**; **Supplementary Table 2**). Previous literature showed that the representative inflammatory cytokine IL-1β was elevated in PWS, but it was not detected by the multi-factor kit. We re-detected IL-1β with Elisa and found in line with previous reports, higher levels of IL-1β were observed in PWS patients compared to the controls (**Figure 1D**). The difference in serum cytokine levels was still significant when the PWS and control groups had comparable BMIs (21.59 ± 6.17 vs 21.43 ± 10.93) indicating that obesity is not a central driver for the difference in serum. In addition, we found that IL-1β and most of the other indicators had no correlation with BMI in the PWS patients (**Figure 1E**, **Supplementary Figure 1**, **Supplementary Table 3**). Based on preliminary results, we speculated thar the deletion of the genes within the 15q11-q13 region, rather than obesity, may be responsible for the increased levels of inflammatory serum markers.

### Unbiased clustering analysis of PBMCs and cell types identification

**Figure 2:**
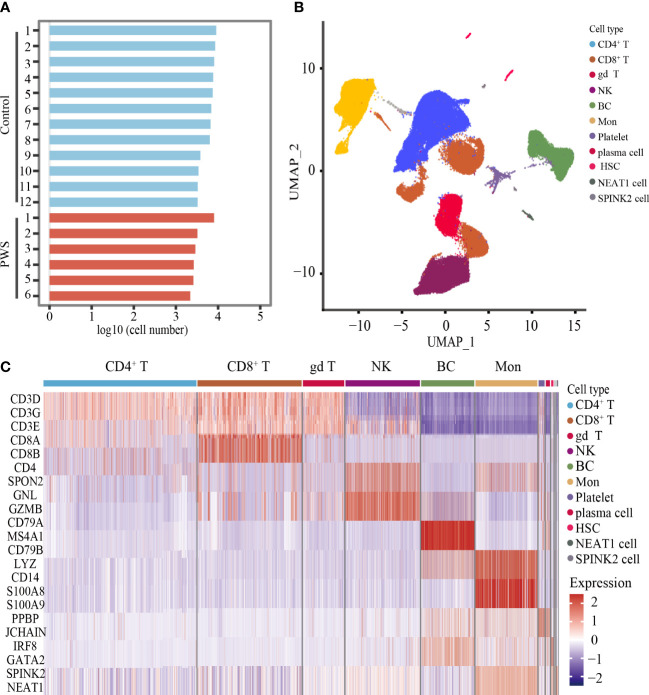
Unbiased clustering analysis of PBMCs and cell types identification. **(A)** Bar plot showing the log10 transformed cell number for each participant. **(B)** UMAP plot of all cells derived from scRNA-seq data. **(C)** Heatmaps showing the expression level of cell type-specific genes for each cluster.

Increased pro-inflammatory cytokines in blood are important markers to reflect the state of activation in immune cells. To better understand the PWS peripheral immune cells, we used the scRNA-seq to examine transcriptome of immune cells in the PWS and control groups. Droplet-based scRNA-seq technology was used to profile PBMCs derived from 6 patients with PWS and 12 healthy controls. The PWS and control group were well matched in terms of age, gender, and BMI (**Supplementary Table 4**). We analyzed a total of 96,067 (control: 74,457; PWS: 21,610) cells in all participants after stringent filtering of the scRNA-seq data (**Figure 2A**), with an average 6,754 UMIs per cell and 2,037 genes per cell. The total number of cells, median genes per cell, and median UMIs per cell were provided in **Supplementary Table 5**. After unbiased clustering analysis, data were then visualized by UMAP and the cellular populations in PBMCs were identified using well-known marker genes (**Figure 2B**; **Supplementary Table 6**). We identified six major immune cell lineages including CD8^+^ T cells, CD4^+^ T cells, gamma delta T cells (gd T), natural killer cells (NK), B cells (BC), and monocytes (Mon) (**Figure 2C**). In downstream analysis, we focused on the six major cell types. No significant differences were found in the number of cells in the six major immune cell lineages between the two groups and only the number of CD4^+^ T cells was marginally elevated (**Supplementary Figure 2**).

### PWS peripheral immune cells were in a hyper-inflammatory state

**Figure 3:**
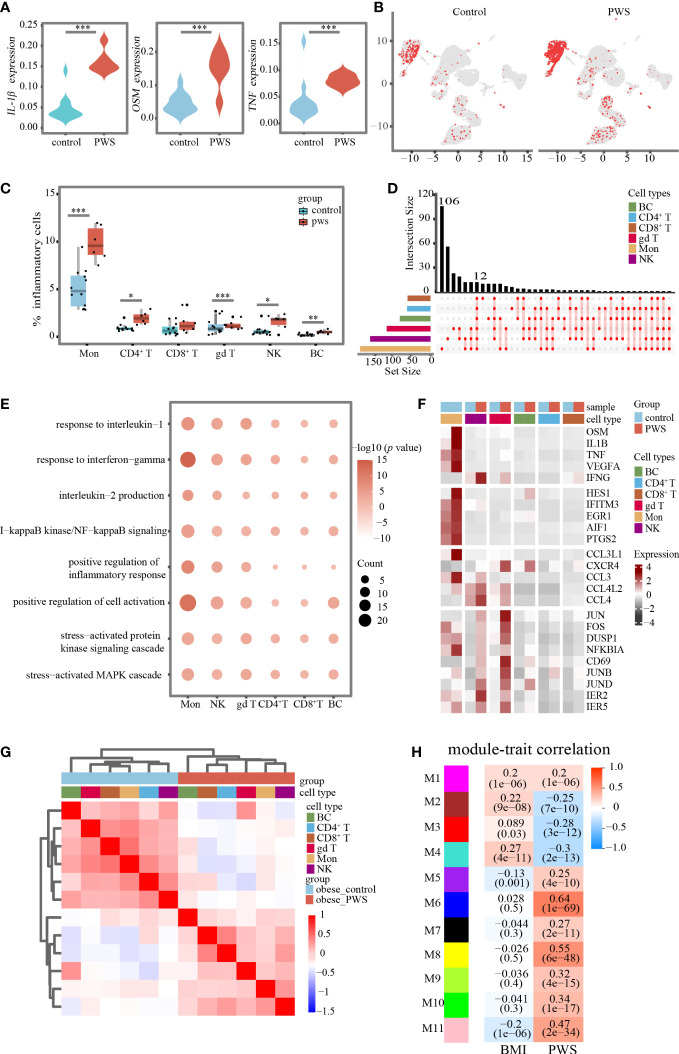
Changes in transcriptional landscape of PBMCs in PWS. **(A)** Differential expression levels of inflammatory-related genes between controls and PWS patients in all cells (Mann-Whitney U test was applied. ***p < 0.05). **(B)** UMAP plots showing inflammatory cells in controls and PWS patients. **(C)** Percentage of inflammatory cells in major cell types of the two groups(Mann-Whitney U test was applied. *p < 0.05, **p < 0.01, ***p < 0.001) **(D)** Comprehensive comparative analysis of upregulated DEGs in major cell types between the PWS and control groups. **(E)** Representative GO terms for upregulated genes in PWS patients compared to controls in each major cell type. **(F)** A heatmap showing the scaled expression of inflammatory-related genes in major cell types. **(G)** Hierarchical clustering was based on by Pearson correlation coefficient (PCC). The intensity of the color represents the PCC values. Color bars above the heatmaps indicate the cell type and the study group. **(H)** Heatmap of the correlation between module eigenvalues and clinical traits. Color of the heatmap indicates correlation coefficient. Numerical values in the brackets indicate correlation coefficient and the *p-*value of the correlation coefficient.

Consistent with above results, transcription levels of *IL-1β*, *TNF*, and *OSM* were globally elevated in the PWS group (**Figure 3A**), yet the source of the cytokines in peripheral immune cells remains unclear. We labeled cells with high expressed pro-inflammatory cytokine genes including IL-1β, TNF and OSM, and named these cells as inflammatory cells. The UMAP plot confirmed an expansion of inflammatory cells in PWS (**Figure 3B**). Globally, we found inflammatory cells increased by approximately 100% in all PBMCs using scRNA-seq analysis (**Supplementary Table 7**). When examining inter-individual variation in the six major populations, we noted that monocytes were the predominant source of inflammatory cells in PWS (**Figure 3C**).

An integrated comparative analysis of DEGs in PBMCs from the PWS and control groups was conducted to identify cell-type-specific gene signatures associated with PWS (**Supplementary Table 8**). A set of 12 genes related to inflammation (e.g., *IER5, JUNB, JUND, NFKBIA, ZFP36*, and *CXCR4*) were found to be upregulated across all major cell types in PWS (**Figure 3D**). Next, we explored the biological implications of upregulated DEGs using the Gene Ontology (GO) pathway analysis for each major cell type. The generally upregulated genes across major cell types were enriched in inflammatory-related pathways, such as the inflammatory response pathway, NFkB signaling pathway, production of inflammatory cytokines, and stress-related pathways (**Figure 3E**; **Supplementary Table 9**). In addition, we have also found PWS-disease-specific inflammatory-related pathways such as aging, cell cycle arrest, and mitotic cell cycle arrest (see **Supplementary Table 9**). The above result revealed that, in PWS, peripheral blood immune cells may be influenced by common inflammatory mediators regardless of cell type. We also observed cell type-specific enriched pathways in PWS such as regulation of neuron death pathway was enriched in natural killer cells, response to starvation in B cells, positive regulation of cell-cell adhesion in monocytes, and toll-like receptor signaling pathway in CD8^+^ T cells (**Supplementary Table 10**).

Differential gene expression analysis revealed that monocytes exhibited the largest number of gene expression changes among the major cell types. Furthermore, the PWS group had significantly higher expression of genes including inflammatory response genes (e.g., *PTGS2, PTGER3* and *ICAM1*), and chemokine (e.g., *CCL2* and *CXCL9*) or cytokine genes (e.g., *IL-1β, OSM* and *TNF*) compared to the control group. Most of these genes had the highest expression value in monocytes (**Figure 3F**). Moreover, in the PWS group, a positive correlation was observed between the *IL-1β* transcription levels in monocytes and serum IL-1β levels (**Supplementary Figure 3A**). A similar result was also observed for the *TNF* transcription levels in monocytes and serum TNF-α levels (**Supplementary Figure 3B**). These results suggested that monocytes were closely related to the development of inflammation in PWS and that the elevation of serum inflammatory cytokines previously reported in the literature may be largely due to activation of the monocytes.

Traditionally, PWS has been considered as an obesity-related disease, we assessed whether observed transcriptional differences could be attributed to obesity. In contrast to conventional views, we found distinct transcriptional signatures between obese PWS and obese controls in major cell types. To visualize overall transcriptome changes, we performed hierarchical clustering. Surprisingly, all major cell types were clustered together according to the study groups instead of cell types (**Figure 3G**). In addition, we performed weighted gene co-expression network analysis to identify modules associated with PWS and BMI (**Figure 3H**). We noted that modules correlated with PWS was not identical to BMI-associated modules which further supporting our hypothesis the observed changes in peripheral immune cells were not mainly due to obesity.

### CD16^+^ monocytes and their role in promoting PWS inflammation in global single-cell profiling

**Figure 4:**
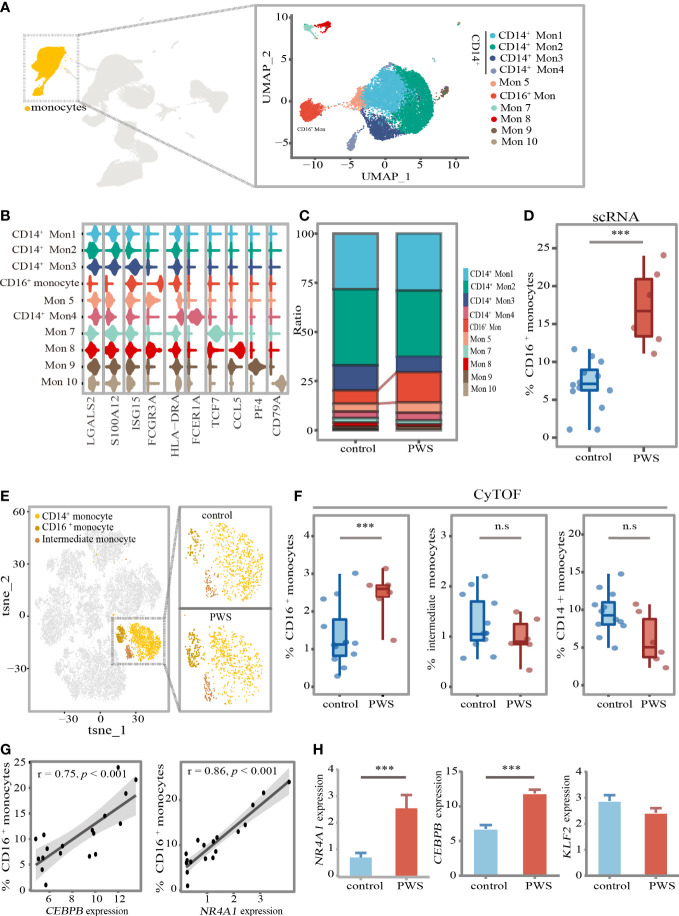
CD16^+^ monocytes increased in PWS patients. **(A)** UMAP visualization of the transcriptional heterogeneity of circulating monocytes. The monocytes are further divided into ten clusters, and their names are annotated on the right. Different colors are used to distinguish each cluster. **(B)** Violin plot showing the signature expression genes of each cell cluster. **(C)** Proportion of cell types in each group. The colors indicate cell types information. **(D)** Percentage of CD16^+^ monocytes (identified by scRNA-seq) in the monocytes of the control and PWS group. *p*-values were defined by the Mann-Whitney U test. ***p<0.05. In the boxplot, each dot represents a sample. Boxes range from the 25th to the 75th percentiles. The upper and lower whiskers extend from the box to the largest and smallest values respectively. **(E)** tSNE representative map of PBMCs clusters derived from PWS patients (n=6) and controls(n=12) by CyTOF and highlighting three monocytes subclusters (dark yellow). **(F)** Percentage of three monocytes subclusters (identified by CyTOF) in PBMCs of the control group and PWS. *p*-values were defined by the Mann-Whitney U test. ***p<0.05, n.s., no significance. **(G)** Spearman correlations between the percent of CD16^+^ monocytes (identified by scRNA-seq) and *NR41A* and *CEBPB* gene expression levels. **(H)** Expression of *NR4A1*, *CEBPB* and *KLFF2* in PWS (n=6) and controls (n=12) of monocytes. *p*-values were defined by the Mann-Whitney U test. ***p<0.05.

The findings presented heretofore indicate that, among the six major cell types, the monocytes were most closely related to the development of inflammation in PWS. In order to further uncover PWS-specific transcriptional signatures in monocytes, we performed sub-clustering analysis of monocytes using Seurat and identified ten clusters according to specific markers (**Figures 4A, B**; **Supplementary Figure 4**-**5**). To describe how cell- type composition changed in PWS of monocytes, we separately compared the percentage of each cluster between the PWS and control groups. CD16^+^ monocytes exhibited the greatest changes and increased by approximately 100% based on the scRNA-seq analysis (**Figures 4C, D**). CD16^+^ monocytes, characterized by high expression of CD16 (FCGR3A) and low levels of CD14, are most closely resembled the well-defined nonclassical monocytes (**Supplementary Figure 4**). To validate the expansion of CD16^+^ monocytes, we performed CyTOF analysis of 6 PWS patients and 12 controls using PBMCs. We identified major cell types in PBMC and three types of monocytes including CD14^+^ monocytes, intermediate monocytes (IM) and CD16^+^monocytes according to cell markers (**Supplementary Figure 6**). Remarkably, CD16^+^ monocytes increased by approximately 80% (**Figures 4E, F**). The increase in CD16^+^ monocytes in PWS may be due to the conversion of other types of monocytes to CD16^+^ monocytes. Previous work has demonstrated that the transcription factor NR4A1 which regulated by KLF2 or CEBPB is the master regulator of the CD16^+^ monocytes (25–27). Similarly, we also found that the number of CD16^+^ monocytes were positively correlated with Nr4a1 and CEBPB (**Figure 4G**). In PWS patients the expression of the genes *NR4A1* and *CEBPB* were elevated while *KLF2* was not affected, which could partially account for the increase of CD16^+^ monocytes in PWS (**Figure 4H**).

**Figure 5:**
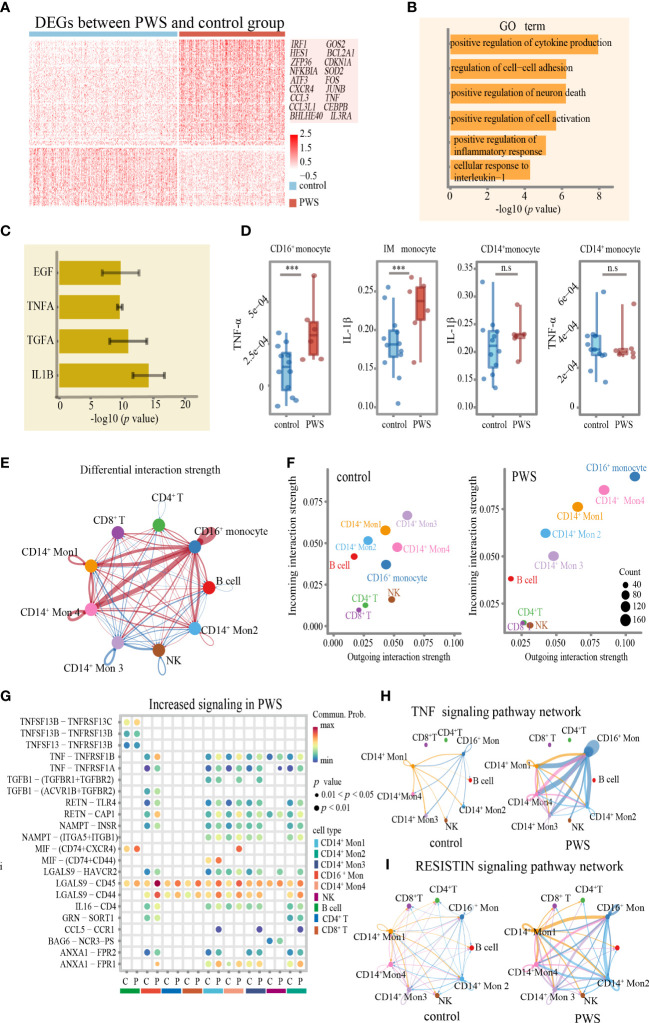
Transcriptome of CD16^+^monocytes in PWS patients. **(A)** Heatmap of differentially expressed genes between PWS patients and healthy controls in the CD16^+^ monocytes. **(B)** Bar plots of GO terms enriched in CD16^+^ monocyte from the PWS patients. **(C)** Bar plots showing the average –log10(*p-value*) values in enrichment analysis using the perturbed genes of different cell lines listed in L1000 LINCS for up-regulated genes in PWS. Error bars indicate standard deviation. **(D)** TNF-α and IL-1β proteomic levels in three monocyte clusters were measured by CyTOF in PWS (n=6) controls (n=12). *p*-values were defined by the Mann-Whitney U test. ***p<0.05, n.s., no significance. **(E)** Circle plot of differential interaction strength in PWS compared to the control group. In the circle plot, red (or blue) indicates increased (or decreased) signaling in PWS compared to the control group. Line thickness represents the interaction strength on a continuous scale (thicker = stronger interaction). **(F)** Scatter plot of incoming and outgoing interaction strength of cell types in the control and PWS groups. **(G)** The bubble plot of the communication probability of some significant ligand-receptor pairs from CD16^+^monocytes to other cell types in PWS. The bubble color gradient and size indicate the communication probability and *p*-values (permutation test), respectively. C, control; P, PWS. **(H, I)** Circle plots of TNF and RESISTIN signaling network in the PWS and control groups. The edge width represents the communication probability. Thicker edge line indicates a stronger signal.

We wondered whether CD16^+^ monocytes not only manifested by increased cellular number but also altered cellular states to elevate levels of inflammation in PWS. Indeed, we identified lots of disease-specific genes expressed genes in CD16^+^ monocytes (**Figure 5A**). A large number of upregulated genes with inflammation-related functions were observed in the CD16^+^ monocytes in PWS, including inflammatory activation-associated genes (e.g., *IRF1*, *HES1*, *NFKBIA*, *ZFP36* and ***ATF3***), and inflammation-related chemokine genes (e.g., *CXCR4*, *CCL3*, and *CCL3L1*). We also found that genes related to aging (e.g., *BHLHE40*, *CDKN1A* and *BCL2A1*), and stress response (e.g., *GOS2* and *SOD2*) were upregulated in PWS (**Supplementary Table 11**). An extended GO analysis of these genes revealed enrichment in pathways mainly involved in the regulation of the inflammatory response and cytokine production (**Figure 5B**; **Supplementary Table 12**). To further understand the biological functions of these genes, we examined the upregulated DEGs by gene set enrichment analysis using cytokine-responsive gene sets from cytokine-treated cells (LINCS L1000). PWS-upregulated DEGs were enriched by TNF/IL-1β- responsive genes (**Figure 5C**) and the result was confirmed by CyTOF. We found that the protein expression of TNF-α in CD16^+^ monocytes was significantly higher in the PWS group than the control group. IL-1β was significantly increased in PWS patients compared to the controls in intermediate monocytes. However, we did not detect increased IL-1β or TNF-α in CD14^+^ monocyte (**Figure 5D**).

Interestingly, in CD16^+^ monocytes pathway analysis showed significant enrichment in the communication between immune cells in PWS (**Figure 5B**). Given that CD16^+^ monocytes might regulate the inflammatory process of other cells though cell–cell interactions, we applied CellChat to infer and analyze the intercellular communication networks to identify the alterations of interactions between CD16^+^ monocytes among other cell types. By comparing the outgoing and incoming signals of cell types in the PWS and control group, we noticed that CD16^+^ monocytes in the PWS group showed greater changes in transmitted and received signaling compared to those in the control group (**Figures 5E, F**) which implied that CD16^+^monocytes may have an increased tendency for interaction with other immune cells in blood vessels. It was notable that the expression of multiple inflammation -related cytokines/receptors were significantly increased in PWS patients such as TNF, RETN, and its receptors, through that CD16^+^ monocytes may interact with the other monocytes (**Figure 5G**). Similarly, increased levels of LGALS9 and its receptor were observed in CD16^+^monocytes of PWS, which suggesting the potential functional interaction of the CD16^+^monocytes with CD8^+^ T cells, CD4^+^ T cells. Chemokines such as MIF, and CCL5 and their respective receptors were also found to be enriched in PWS CD16^+^monocytes. Correspondingly, we also found that CD16^+^ monocytes of the PWS group showed more output related to TNF-α signaling and VISFATIN signaling (**Figures 5H, I**). Overall, these results help illustrate the possible molecular basis for communication between peripheral immune cells of PWS patient leading to a better understanding of the mechanisms about elevated levels of inflammation in PWS.

### The 15q11–q13 region plays a critical role in regulating the peripheral immune cells inflammation

**Figure 6:**
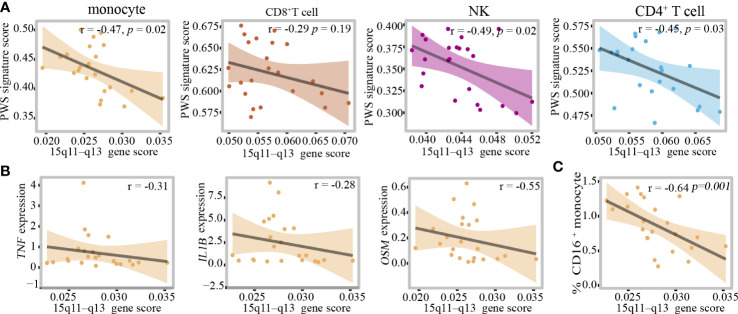
The 15q11–q13 region plays a critical role in regulating the peripheral immune cells inflammation. **(A)** Pearson correlation between PWS signature scores and 15q11–q13 gene scores in the healthy group. **(B)** Spearman correlation between 15q11–q13 gene scores and proinflammatory genes expression level in the healthy group. **(C)** Spearman correlation between 15q11–q13 gene scores and the percent of CD16^+^monocytes in the healthy group.

In monocytes, there was no correlation between proinflammatory cytokines and BMI, so we hypothesized that the 15q11–q13 region might be responsible for elevated levels of inflammation. Due to the limited number of healthy controls in this study, we created a new healthy control group which comprised by two groups, one from the control group in this study consisting of 12 healthy individuals and another from our unpublished study consisting of 11 healthy individuals. Additional demographic data are provided in **Supplementary Table 13**. To assess the impact of the 15q11–q13 region on circulating immune cells, we selected the genes of the 15q11–q13 region and calculated 15q11–q13 gene scores in each cell using AUCell to evaluate gene expression in the 15q11–q13 region in the healthy group. We also calculated the score of the PWS- related transcriptome in the healthy group using the up DEGs in PWS called PWS signature scores (**Supplementary Table 14**). Surprisingly, there was a negative correlation between the PWS signature scores and 15q11–q13 gene scores in the healthy group (**Figure 6A**). This result implies that the healthy individuals with lower 15q11–q13 scores tend to display PWS-related transcriptional changes. Furthermore, in the healthy group, negative associations were observed for *IL-1β*, *TNF*, *OSM*, and 15q11–q13 gene scores (**Figure 6B**) indicating that healthy individuals with low expression of 15q11–q13 gene prone to express more pro-inflammatory cytokines. There was also a negative correlation 15q11–q13 gene scores and percentage of CD16^+^ monocytes in the healthy group (**Figure 6C**).

In order to quantified the individual levels of inflammation, we used inflammation-related gene set which was obtained from Molecular Signatures Database Hallmark to calculate individual inflammation scores. Additionally, 15q11–q13 gene scores were associated with inflammation scores in the healthy individuals after adjusting for the relevant confounders such as BMI and age (**Table 1**). The regression analysis revealed that 32.6% of the variance in inflammation scores was explained by15q11–q13 gene scores. The results, showed that the 15q11–q13 region affected the overall level of inflammation in healthy person. Next, we made a further analysis to determine if the relationship between 15q11–q13 region gene and inflammation was mediated by CD16^+^ monocytes by the R process mediation test. Mediation analysis revealed that both the total and indirect effects (ACME *p <*0.05) of the 15q11–q13 region scores on the inflammation scores were significant; however, the direct effect of the 15q11–q13 region on the inflammation scores was insignificant (ADE *p* > 0.05) (**Table 2**). The findings indicated that the lower 15q11–q13 gene expression observed in healthy individuals with a tendency toward higher inflammation score may be explained at least in part by high CD16^+^ monocytes levels. Collectively, the findings revealed a previously under-appreciated link between 15q11–q13 region peripheral and immune cells inflammation which might provide a theoretical basis for the gene therapy or immunotherapy in PWS patients.

**Table 1:** Multiple linear regression analysis to assess influence of variables on inflammation scores.

inflammation score					
Model	B	*P*	95% CI	R^2^	*P*
PWS score	-0.460	0.017	-2.179 -0.239	0.326	0.015
BMI (kg/m2)	-0.337	0.110	-0.001 0.000		
Age(years)	-0.110	0.590	-0.003 0.002		

ß, linear regression coefficient; CI, Confidence interval.

**Table 2:** The mediation analysis of CD16^+^monocytes in association between 15q11–q13 gene scores and inflammation score.

	Estimate	95% CI Lower	95% CI Upper	*P*
ACME	-0.64	-1.56	-0.01	<0.05
ADE	-0.70	-1.81	0.44	0.22
Total Effect	-1.34	-2.37	-0.24	<0.05

ACM, Average causal mediation effects, namely, indirect effect; ADE, Average direct effects, namely, direct effect.

## Discussion

In the present study, we first provide insights into the PBMCs of PWS patients at single-cell resolution. We identified the major cell types in PBMCs and elucidated their contributions to inflammation. We found that monocytes, especially the CD16^+^ monocytes, had a key role in promoting proinflammatory activities and network in PWS. Moreover, we showed that loss of gene expression from the chromosome 15q11–q13 locus was closely related to inflammation in healthy people.

Multiple studies have shown that PWS confers a relative risk for inflammatory-related diseases including Type 2 diabetes mellitus (T2DM), cardiovascular diseases and mental diseases compared with non-syndromic obesity. It has been reported that T2DM is common in PWS (8) and more than 50% of the PWS patients have developed diabetes before the age of 18 (28, 29). Furthermore, subtle atherosclerosis starts in young patients with PWS has also been demonstrated (10). A number of mental disorders have been widely reported in patients with PWS (30). Importantly, although rare, there were several studies reported that serum levels of circulating inflammatory markers were increased in PWS. Similar to previous reports, pro-inflammatory cytokines such as IL-1β, IL-16, IL-18 and TNF-α which were predominantly derived from activated immune cell were increased in PWS patients according to the study. Notably, the transcriptional levels of IL-1β and the serum concentrations of IL-1β were both significantly increased in PWS. There have been many reports that IL-1β plays a central role in mediating DM, cardiovascular diseases, and progression of mental disorders. In atherosclerotic coronary arteries, IL-1β levels were correlated with disease severity and knocking out IL-1β in atherosclerosis-prone ApoE−/− mice led to attenuation of disease development (31). IL-1β is believed to impair islet function and viability by activation of the inflammasomes in islet inflammatory cells (32). A separate study implied that subclinical inflammation, observed as elevated IL-1β and IL-13 levels, was correlated to several psychopathological symptoms in PWS (14). Thus, we speculated that PWS comorbidities that may be mediated, in part, by immune activation to induce the production of various proinflammatory cytokines. To yield convincing results, clinical trials about the relationship between PWS pro-inflammatory phenotype and clinical presentation need to be done in the future.

We found that there was no significant correlation between most of cytokines and BMIs in PWS patients which suggested that the pro-inflammatory state in PWS patients cannot be attributed totally to overweight or obesity. Nevertheless, in healthy population, negative associations were observed between IL-1β, TNF, OSM, and 15q11–q13 gene scores. In the conventional view, PWS disease is one of the obesity-related diseases. However, according to our results, obesity is unlikely to be the only factors that influence PWS inflammatory status, a more plausible scenario is that PWS hyper-inflammatory state results from the interplay among deletion of genes, obesity and other factors. However, it remains to be determined which of genes in 15q11–q13 region that involved in progression and development of inflammation in PWS.

Increased circulating inflammatory cytokines potentially reflected the alteration of the inflammation profile of peripheral immune cells in PWS. Therefore, we applied scRNA-seq and CyTOF to explore the changes in peripheral immune cells. we found that CD16^+^ monocytes were significantly increased in PWS. Traditionally, according to the CD14 and CD16 expression patterns, peripheral blood monocytes are divided into three types: classical, intermediate, and nonclassical monocytes. We showed that CD14^+^ Mon1-4 appeared most analogous to classical monocytes. Based on numerous studies using scRNA-seq, monocytes that express high levels of CD16 and low levels of CD14 are commonly grouped into a single cluster, which is consistent with our findings (**Supplementary Figure 4**). This cluster is typically referred to as CD16^+^ monocytes (33–35). Researchers assumed that CD16^+^ monocytes most closely resembled the well-defined nonclassical monocytes (35–38). Mon 5 did not form a distinct population but was mainly distributed at the junction between CD14^+^ monocytes and CD16^+^ monocytes were more like intermediate monocytes. Few monocytes shared discriminative genes with megakaryocytes, such as PPBP and PF4 and several monocytes expressed the gene signature of NK cells (e.g., high expression levels of FGFBP2, GNLY, GZMA, and IL32), were also identified (**Supplementary Figure 5**). This illustrates the advantage of the scRNA-seq which could characterize human monocyte clusters in unprecedented detail.

Nr4a1 is necessary for nonclassical monocytes generation and development as Nr4a1-/- mice lack nonclassical monocytes. Graham D et al. identified Klf2 can regulate nonclassical monocyte conversion *via* cell-specific super-enhancer domain E2.The E2 is a single sub-domain (E2) 4 kb upstream of the Nr4a1 transcription start site and was essential for nonclassical monocytes development (26). In Lyz2-cre Klf2^flox/flox^ mice Ly6C^hi^ monocytes were unaffected however Ly6C^low^ nonclassical monocytes were partially reduced. Expression of the monocyte survival factor Nr4a1 is also regulated by C/EBPb which could regulate monocyte differentiation into nonclassical monocyte. In PWS patients the expression of the genes *NR4A1* and *CEBPB* were elevated while *KLF2* was not affected which could partially account for the increase of CD16+ monocytes in PWS.

CD16^+^ monocytes most closely resembled the well-defined nonclassical monocytes, are usually referred to as anti-inflammatory cells. Nevertheless, it was recently shown that CD16^+^ monocytes are also capable of exerting proinflammatory responses depending on the disease context. Ratnadeep Mukherjee demonstrated that CD16^+^ monocytes were the primary producers of TNF-α and IL-1β upon *ex-vivo* activation of whole blood with lipopolysaccharides (39). The observed phenomena are explained by high basal levels of phosphorylated NF-κB in CD16^+^ monocytes which is a transcription factor for pro-inflammatory cytokines (40). Moreover, CD16^+^ monocytes enter the peripheral tissues and differentiate into inflammatory macrophages (M1), and are involved in regulating inflammation in peripheral tissue (41). Clinical data showed that CD16^+^ monocytes were increased in various inflammatory conditions, such as coronary artery disease (42), liver fibrosis (43), NAFLD (44), aging (40), and systemic sclerosis (45). CD16^+^ monocytes are thought to contribute to the chronic inflammation associated with these diseases, and they may also be involved in the destruction of healthy tissues. Thus, we hypothesized that CD16^+^ monocytes may have proinflammatory and anti-inflammatory functions, with a greater inclination towards anti-inflammatory functions under normal conditions. However, in certain disease contexts, the balance of pro-inflammatory and anti-inflammatory functions in CD16^+^ monocytes may become disrupted, leading to an increased expression of pro-inflammatory properties. We also found that PWS CD16^+^ monocytes upregulated DEGs were enriched by TNF/IL-1β- responsive genes. We further clarified their role in promoting PWS inflammation at global single-cell profiling. In addition, we made a further analysis to determine the relationship between 15q11–q13 region gene and inflammation was mediated by CD16+ monocytes by the R process mediation test (**Table 2**). Given lots of changes displayed by CD16^+^monocytes in PWS, and the previously reported etiological link between CD16^+^monocytes activity and inflammation-related diseases, we put forward a plausible view that the changes in CD16^+^ monocytes state participated in hyper-inflammatory phenotype and comorbidities of PWS.

Despite some important findings made in this study, article had several limitations. First, as a result of the limited number of PWS patients enrolled in our study, the differences we identified between PWS patients and controls need to be future validated by larger clinical trials. Second, since it was a single time point study, drawing causal conclusions was not possible. The study did not provide evidence as to whether CD16^+^ monocytes are associated with the severity of inflammatory diseases in PWS.

In conclusion, based on an unbiased scRNA-seq approach, we established an atlas of PWS circulating immune cells and offered insights into the function of CD16^+^ monocytes which partially contributed to a hyper-inflammatory state in PWS. Further study is needed to elucidate the mechanism underlying the CD16^+^monocytes and its broader implications, and thus help identify novel therapeutic target for PWS patients.

## Data availability statement

The sequencing data has been deposited in GSA-Human (https://ngdc.cncb.ac.cn/) under the accession number HRA003585.

## Ethics statement

The studies involving human participants were reviewed and approved by the Ethics Committee of Shandong Provincial Hospital. Written informed consent to participate in this study was provided by the participants’ legal guardian/next of kin.

## Author contributions

JZ, YFS, LG conceived the study. YX, HG, XF and ZY performed most of the bioinformatics analysis and wrote the manuscript. GL, YW, XH, CX and YS collected the samples. All authors contributed to the article and approved the submitted version.
