# Supplementary material for: CD16+ monocytes are involved in the hyper-inflammatory state of Prader-Willi Syndrome by single-cell transcriptomic analysis
Source: Front Immunol. 2023 May 11;14:1153730. doi: 10.3389/fimmu.2023.1153730 (PMC10213932; doi:10.3389/fimmu.2023.1153730)
Supplement: Supplementary file 1 [file DataSheet_1.zip › Supplementary material/Supplementary Table 13.docx]

**Supplementary Table 13** General demographic information

|  | n | Mean | Median | SD | Minimum | Maximum |
| --- | --- | --- | --- | --- | --- | --- |
| 8 male/ 15 female |  |  |  |  |  |  |
| Age (years) | 23 | 8.26 | 9.00 | 1.23 | 6.00 | 11.00 |
| BMI (kg/m²) * | 23 | 22.72 | 20.00 | 7.11 | 13.96 | 36.40 |
