# Supplementary material for: CD16+ monocytes are involved in the hyper-inflammatory state of Prader-Willi Syndrome by single-cell transcriptomic analysis
Source: Front Immunol. 2023 May 11;14:1153730. doi: 10.3389/fimmu.2023.1153730 (PMC10213932; doi:10.3389/fimmu.2023.1153730)
Supplement: Supplementary file 1 [file DataSheet_1.zip › Supplementary material/Supplementary Table 4.docx]

**Supplementary Table 4** General demographic information

|  | control | PWS | *p* |
| --- | --- | --- | --- |
| N (male/female) | 12(9/3) | 6(6/0) | >0.05 |
| Age (years) | 8.25±0.57 | 5.33±1.41 | >0.05 |
| BMI (kg/m2) | 21.91±1.83 | 20.79±4.29 | >0.05 |

All values are represented as means ± standard error of the mean. The sex difference between two group was evaluated with the chi-square test (Fisher’s exact test), Student’s t test was used to the evaluation of remaining variables.
