# Supplementary material for: CD16+ monocytes are involved in the hyper-inflammatory state of Prader-Willi Syndrome by single-cell transcriptomic analysis
Source: Front Immunol. 2023 May 11;14:1153730. doi: 10.3389/fimmu.2023.1153730 (PMC10213932; doi:10.3389/fimmu.2023.1153730)
Supplement: Supplementary file 1 [file DataSheet_1.zip › Supplementary material/Supplementary Table 5.docx]

**Supplementary Table 5** Total number of cells, median genes and median UMIs per cell

| Total number of cells, median genes and median UMIs per cell | | | |
| --- | --- | --- | --- |
| group | Number of cells | median genes per cell | median UMIs per cell |
| control1 | 8083 | 2376 | 7492.85 |
| control2 | 6343 | 2276 | 7229.14 |
| control3 | 6922 | 2350 | 7763.01 |
| control4 | 9050 | 2153 | 6785.58 |
| control5 | 7479 | 2016 | 6524.02 |
| control6 | 8511 | 1770 | 5661.79 |
| control7 | 3797 | 2012 | 6204.55 |
| control8 | 6610 | 1860 | 5370.35 |
| control9 | 3281 | 2030 | 6207.14 |
| control10 | 3269 | 2008 | 5945.66 |
| control11 | 3429 | 1964 | 6316.58 |
| control12 | 7683 | 1731 | 5414.00 |
| pws1 | 2643 | 1970 | 7879.66 |
| pws2 | 2891 | 2112 | 7419.43 |
| pws3 | 8057 | 1898 | 6080.40 |
| pws4 | 2183 | 1981 | 7854.67 |
| pws5 | 2596 | 2043 | 7661.51 |
| pws6 | 3240 | 2115 | 7777.25 |
