# Supplementary material for: CD16+ monocytes are involved in the hyper-inflammatory state of Prader-Willi Syndrome by single-cell transcriptomic analysis
Source: Front Immunol. 2023 May 11;14:1153730. doi: 10.3389/fimmu.2023.1153730 (PMC10213932; doi:10.3389/fimmu.2023.1153730)
Supplement: Supplementary file 1 [file DataSheet_1.zip › Supplementary material/Supplementary Table 6.docx]

**Supplementary Table 6 Markers for each cell type**

| Defining scRNA markers | |
| --- | --- |
| cell types | Markers |
| CD4^+^ T | CD3D CD3E CD4 |
| CD8^+^ T | CD3D CD3E CD8 |
| gd T | CD3D CD3E CD4- CD8- |
| NK cell | CD3D- GNLY GZMB SPON2 |
| B cell | CD19 MS4A1 |
| monocyte | LYZ CD14 FCGR3A |
| Plasma cell | JCHAIN IGHA1 IGHG1 |
| HSC | GATA2 |
| Platelet | PPBP |
| NEAT1_cell | NEAT1 |
| SPINK2_cell | SPINK2 |
