# Supplementary material for: CD16+ monocytes are involved in the hyper-inflammatory state of Prader-Willi Syndrome by single-cell transcriptomic analysis
Source: Front Immunol. 2023 May 11;14:1153730. doi: 10.3389/fimmu.2023.1153730 (PMC10213932; doi:10.3389/fimmu.2023.1153730)
Supplement: Supplementary file 1 [file DataSheet_1.zip › Supplementary material/Supplementary Table 7.docx]

**Supplementary Table 7** Percentage of inflammatory cells in major cell types of the two groups

|  | Control(n=12) | PWS(n=6) | *p* |
| --- | --- | --- | --- |
| Monocytes (%) | 43.10(26.45-57.01) | 84.34(77.66-86.33) | <0.001 |
| CD4^+^T cell (%) | 3.26(2.14-3.83) | 4.94(4.00-8.45) | 0.02 |
| CD8^+^T cell (%) | 3.19(1.57-4.78) | 5.56(3.56-9.75) | 0.07 |
| NK cell (%) | 3.78(2.42-5.92) | 12.03(7.70-16.34) | 0.01 |
| B cell (%) | 1.90(1.23-2.52) | 4.98(4.26-5.58) | <0.001 |
| gd cell (%) | 11.49(8.69-15.30) | 19.41(17.95-26.05) | <0.001 |

Data are shown as median (interquartile). The p-values comparing the PWS and control are from the Mann-Whitney U test.
