# Supplementary material for: CD16+ monocytes are involved in the hyper-inflammatory state of Prader-Willi Syndrome by single-cell transcriptomic analysis
Source: Front Immunol. 2023 May 11;14:1153730. doi: 10.3389/fimmu.2023.1153730 (PMC10213932; doi:10.3389/fimmu.2023.1153730)
Supplement: Supplementary file 1 [file DataSheet_1.zip › Supplementary material/Supplementary material.docx]

***Supplementary Material***

**Supplementary Figures**


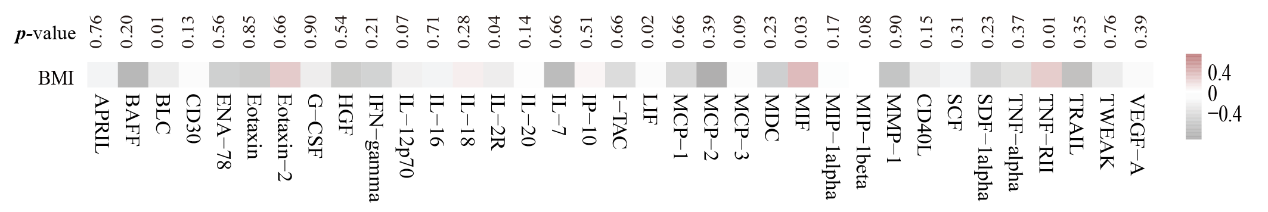


**Supplementary Figure 1.** Correlation between BMI and cytokine levels in PWS. Correlation values are presented in a heatmap (Spearman correlation test). Red represents positive correlation, black represents negative correlation, the darker the color, the stronger the correlation.


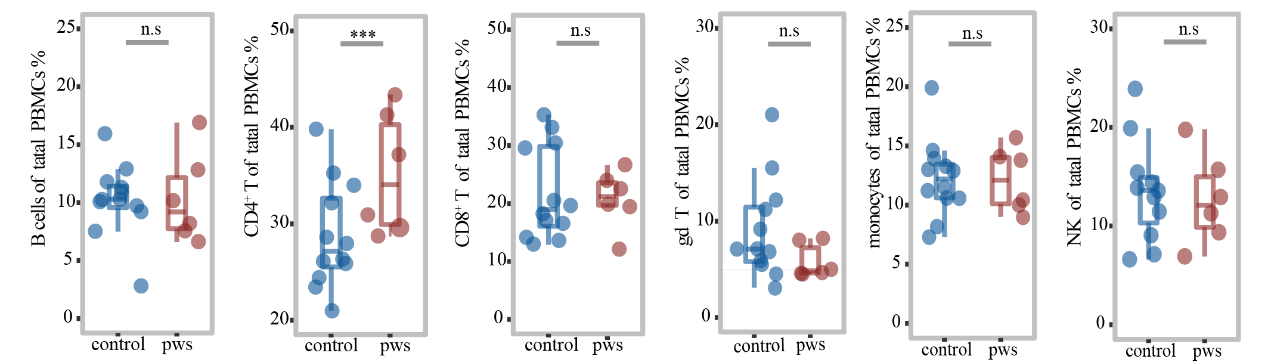


**Supplementary Figure 2.** Major cell type composition in controls and PWS patients.

Dot plots of six major cell types ratios in the PWS groups compared with control group based on scRNA-seq. *p*-values were defined by the Mann-Whitney U test. ****p* <0.05, n.s.: no significance.


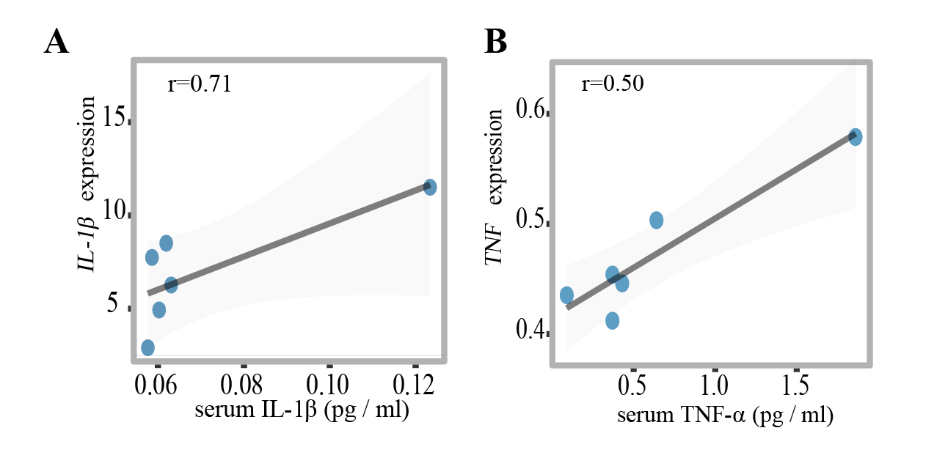


**Supplementary Figure 3.** Serum cytokines levels were correlated with the corresponding transcript levels in monocytes. **(A)** Spearman Correlation between expression levels of transcripts *IL-1β* and serum IL-1β levels in PWS patients. **(B)** Spearman Correlation between expression levels of transcripts *TNF* and serum TNF-α levels in PWS patients.


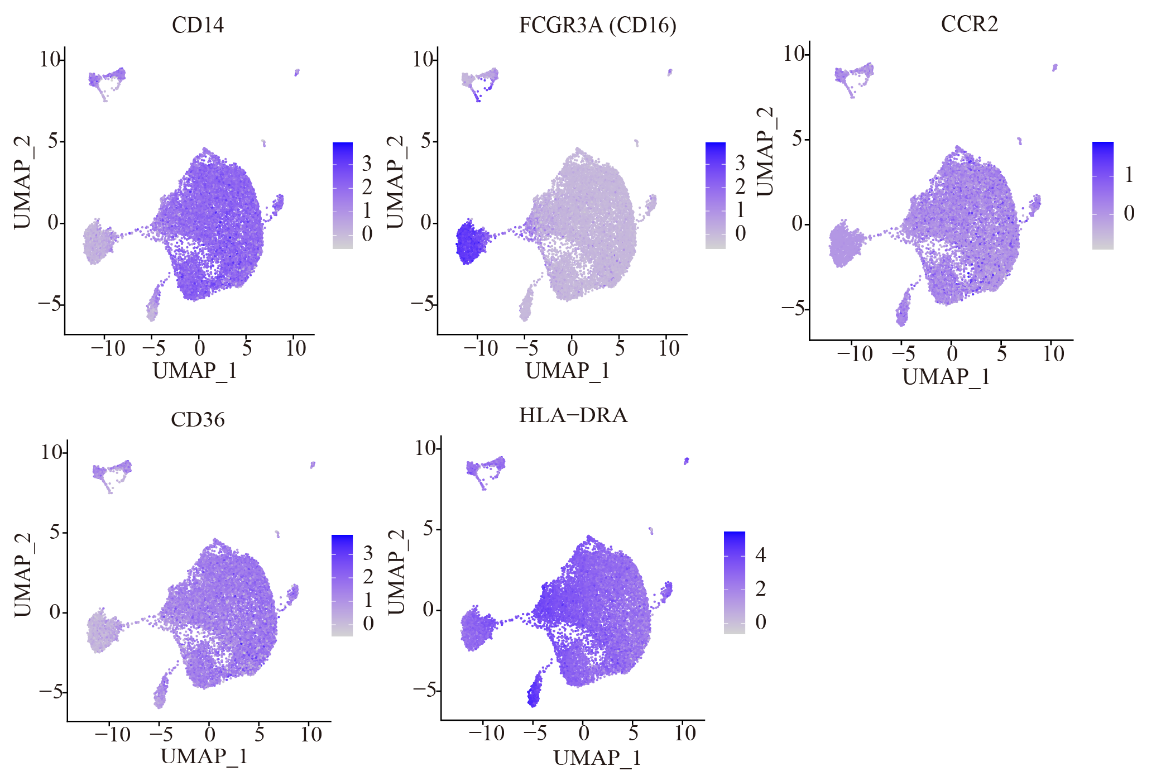


**Supplementary Figure 4.** **Expression of marker genes for monocyte clusters.**

Expression pattern of five marker genes of monocytes. Purple is used to represent the expression of these marker genes, and each dot represents an individual cell.


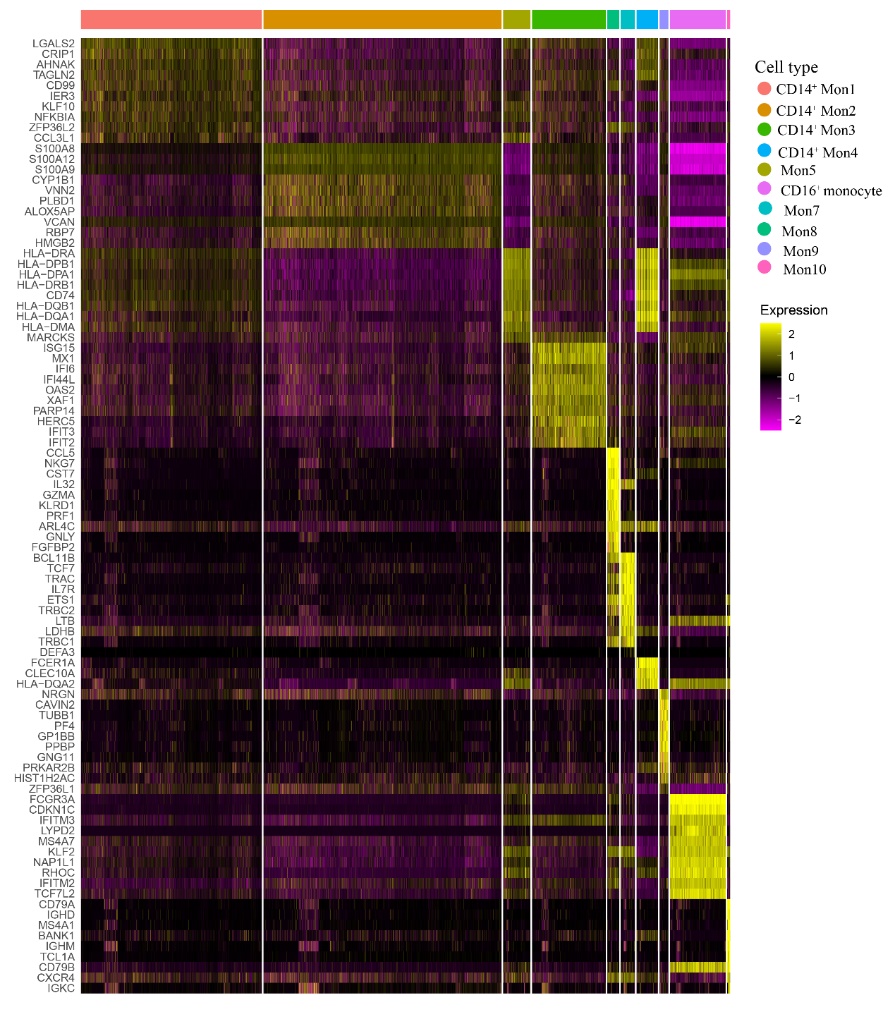


**Supplementary Figure 5. Heatmap of marker genes for the monocyte clusters.**

There are 10 discriminating genes listed for each cluster on the heatmap. The color scheme is based on z-score distribution from −2.5 (purple) to 2.5 (yellow).


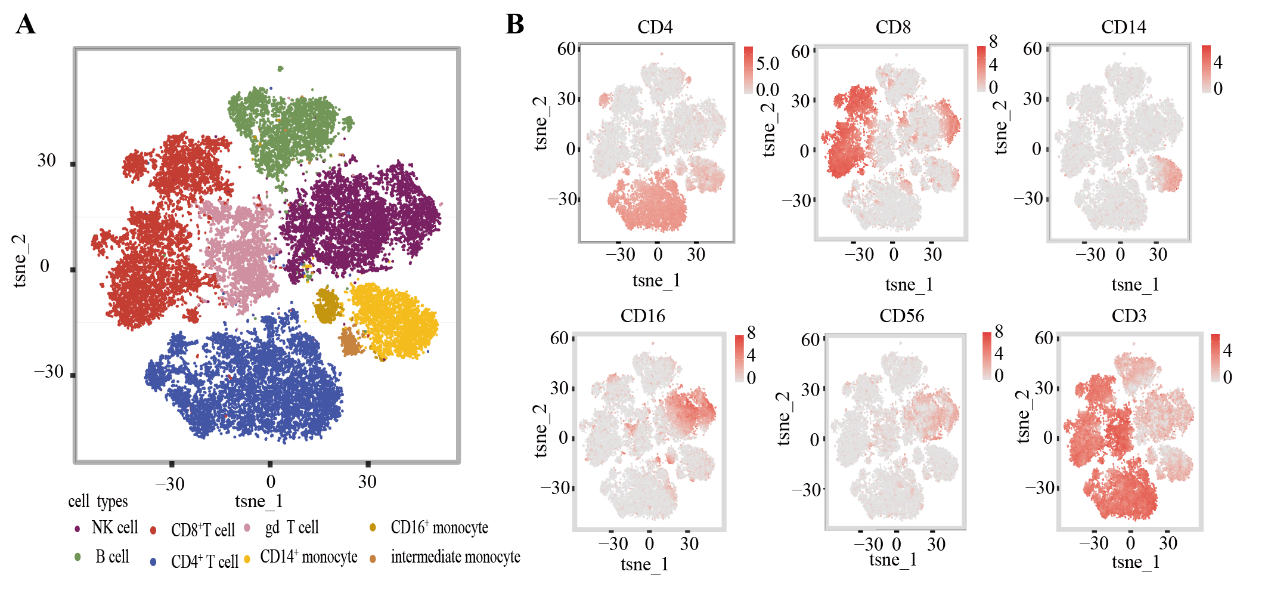


**Supplementary Figure 6.** Unbiased clustering analysis of PBMCs and cell types identification by CyTOF. **(A)** Identified cell types from PBMCs of the control and PWS groups by mass cytometry. **(B)** Normalized intensity of distinct protein markers shown in tSNE visualization that define the cell types by mass cytometry.
